# Spatial and temporal changes in leaf coloring date of *Acer palmatum* and *Ginkgo biloba* in response to temperature increases in South Korea

**DOI:** 10.1371/journal.pone.0174390

**Published:** 2017-03-27

**Authors:** Chang-Kyun Park, Chang-Hoi Ho, Su-Jong Jeong, Eun Ju Lee, Jinwon Kim

**Affiliations:** 1 School of Earth and Environmental Sciences, Seoul National University, Seoul, Republic of Korea; 2 School of Environmental Science and Engineering, South University of Science and Technology of China, Shenzhen, China; 3 School of Biological Sciences, Seoul National University, Seoul, Republic of Korea; 4 Department of Atmospheric and Oceanic Sciences, University of California Los Angeles, Los Angeles, California, United States of America; INRA - University of Bordeaux, FRANCE

## Abstract

Understanding shifts in autumn phenology associated with climate changes is critical for preserving forest ecosystems. This study examines the changes in the leaf coloring date (LCD) of two temperate deciduous tree species, *Acer palmatum* (*Acer*) and *Ginkgo biloba* (*Ginkgo*), in response to surface air temperature (*Ts*) changes at 54 stations of South Korea for the period 1989–2007. The variations of *Acer* and *Ginkgo* in South Korea are very similar: they show the same mean LCD of 295th day of the year and delays of about 0.45 days year^-1^ during the observation period. The delaying trend is closely correlated (correlation coefficient > 0.77) with increases in *Ts* in mid-autumn by 2.8 days °C^-1^. It is noted that the LCD delaying and temperature sensitivity (days °C^-1^) for both tree species show negligible dependences on latitudes and elevations. Given the significant LCD-*Ts* relation, we project LCD changes for 2016–35 and 2046–65 using a process-based model forced by temperature from climate model simulation. The projections indicate that the mean LCD would be further delayed by 3.2 (3.7) days in 2016–35 (2046–65) due to mid-autumn *Ts* increases. This study suggests that the mid-autumn warming is largely responsible for the observed LCD changes in South Korea and will intensify the delaying trends in the future.

## Introduction

Changes in vegetation phenology in spring and autumn are noticeable dynamic responses of the ecosystem to climate change [1, 2, 3]. During the growing season, defined as the difference between the spring and autumn phenological events, vegetation regulates the exchange of carbon, water, and energy between the land and the atmosphere [4, 5, 6]. It is clear that changes in the growing season influence the functioning of ecosystem and biodiversity by modifying the distribution of vegetation populations and interspecies interactions [7, 8, 9]. Hence, understanding the changes in vegetation phenology is a critical step for diagnosing ecosystem changes related to climate changes.

The advancing trend of spring phenology (e.g., first flowering, budburst, and green leafing) in response to warming in winter and spring has been well documented from local to global scale [10, 11, 12, 13, 14]. In contrast, knowledge on the autumn phenology, such as leaf coloring, leaf senescence, and leaf fall, is limited due to the lack of past studies [15]. In particular, the phenological properties of leaf coloring process is hard to identify because of difficulties in observing such processes [16]. Nevertheless, some recent studies indicated that the autumn phenology plays a decisive role in the extension of the growing season in Northern hemispheric temperate forests [17, 18]. For carbon sequestration, the enhancement of vegetation respiration associated with autumn warming may reduce the net carbon fixation [19, 20], implying that the changes in autumn phenology can be more important in future climate conditions.

Although exact factors that control autumn phenology remain uncertain [21], it is known that some climate factors such as photoperiod and temperature, can affect the timing of autumn phenological events [16, 22, 23]. Several literatures pointed out that photoperiod provides the important role in determining the timing of leaf senescence and coloring, especially in the region experiencing severe winter [24, 25]. Temperature variations are closely correlated with the shifts in autumn phenology, both spatially and temporally; thus, have been receiving more attention than photoperiod. Observations in Europe and North America show that temperature increases in late summer and/or autumn affect the delaying trend of the autumn phenology [6, 15, 16, 26, 27]. Various kinds of process-based models have been employed for simulating leaf senescence and coloring for the mid-latitudes temperate tree species using temperature and photoperiod as main drivers, and suggested that such delaying trend in Europe and North America will continue in the future coincide with continued warming [28, 29, 30].

The Intergovernmental Panel on Climate Change (IPCC) Fifth Assessment Report (AR5) showed that East Asian countries have and will experience unfamiliar changes in the terrestrial systems due to substantial surface warming [31]. It is reported that the delaying trend of autumn phenology in East Asia, which is speculated to be occurred by autumn warming, could more dominantly regulate the growing season compared to Europe [32]. Also, it is noted that the temperature sensitivity of autumn phenology in East Asia varies according to environmental conditions of habitat and/or species-specific responsiveness. For example, *Melia azedarach* and *Ulmus pumila* in China showed no significant latitudinal patterns of temperature sensitivity [33], while *Acer palmatum* and *Ginkgo biloba* in Japan indicated apparent negative slope with latitude [34]. Thus, specific information on how autumn phenology responds to warming trends within the framework of each individual region and its native species is essential. However, knowledge on the autumn phenology in East Asia is limited due to a small number of existing studies compared to Europe and North America [35] because of scarce long-term phenological data and/or the lack of research interests [36].

To advance the understanding of autumn phenology in East Asia, this study aims to investigate and project the long-term changes in the leaf coloring dates (LCD) of two native deciduous tree species, *Acer palmatum* (*Acer*) and *Ginkgo biloba* (*Ginkgo*), in South Korea in association with the variations in surface air temperature (*Ts*). Variations of the changes in LCD following latitudes and elevations for the two tree species are analyzed with changing trend of *Ts*. We also apply a temperature-photoperiod model of Jeong and Medvigy [30] in conjunction with a regional climate model, to check the relationship between LCD and *Ts* and project those future changes. Although the location of South Korea is eastern edge of Asian continent, we expect that the results of this study will provide meaningful information on how the autumn phenology of Asian deciduous tree species responds to climate change.

## Materials and methods

### Datasets and analysis methodology

We used the LCD of the two tree species and the *Ts* dataset recorded at 54 weather stations in South Korea that have missing values of LCD for less than three years for the period 1989–2007 (Table 1). The Korea Meteorological Administration (KMA) planted *Acer palmatum* and *Ginkgo biloba* at individual weather station sites and designated them as the standard trees for observing autumn phenology. LCD is recorded when 20% of the leaves of the standard observation trees are colored red or yellow from green. *Ts* is measured using a thermometer positioned 1.5 m above the earth's surface.

**Table 1:** Geographical information and the number of records of 54 weather stations.

Station	Latitude	Longitude	Elevation	Records	Records
Number			(unit: m)	(Acer)	(Ginkgo)
095	38.15	127.30	154.81	17	17
101	37.90	127.73	75.64	19	19
105	37.75	128.88	26.00	19	19
108	37.57	126.95	85.80	19	19
112	37.48	126.62	68.20	19	19
119	37.27	126.98	34.10	17	18
127	36.97	127.95	116.3	19	19
129	36.78	126.50	28.90	18	18
130	36.98	129.42	50.00	19	19
131	36.63	127.43	57.16	17	19
133	36.37	127.37	68.90	19	19
135	36.22	127.98	243.7	18	18
136	36.57	128.70	140.1	19	19
138	36.03	129.38	2.30	19	19
140	36.00	126.75	23.20	18	19
143	35.88	128.62	53.40	19	19
146	35.82	127.15	61.40	19	19
152	35.55	129.32	83.20	18	18
155	35.17	128.57	37.60	19	19
156	35.17	126.88	72.40	19	19
159	35.1	129.03	69.60	18	18
162	34.85	128.43	32.30	18	18
165	34.82	126.38	38.00	19	19
168	34.73	127.73	64.60	18	19
192	35.15	128.03	30.20	18	18
201	37.70	126.45	47.00	18	18
202	37.48	127.50	47.90	18	19
211	38.07	128.17	200.20	19	19
212	37.68	127.88	140.91	18	18
221	37.15	128.18	259.80	18	19
226	36.48	127.73	175.00	19	19
232	36.77	127.12	81.50	19	19
235	36.33	126.55	15.50	19	19
236	36.27	126.92	11.30	19	19
238	36.10	127.48	170.40	19	19
243	35.72	126.70	12.00	19	19
244	35.60	127.28	247.90	18	18
245	35.55	126.87	44.60	19	18
247	35.40	127.33	90.30	19	19
248	35.65	127.52	406.5	19	19
256	35.07	127.23	48.80	19	19
260	34.68	126.92	45.00	17	17
261	34.55	126.57	13.00	19	19
262	34.62	127.28	53.10	19	19
271	36.93	128.92	324.30	17	18
272	36.87	128.52	210.80	19	19
273	36.62	128.15	170.60	18	17
278	36.35	128.68	81.80	19	19
279	36.12	128.32	48.80	19	19
281	35.97	128.95	93.80	18	18
285	35.55	128.17	32.00	19	19
288	35.48	128.75	11.20	19	19
289	35.40	127.87	138.10	19	19
294	34.88	128.60	45.40	17	17
			Total	999	1005

The future *Ts* in South Korea was generated by dynamical downscaling of the global climate projection data from the Community Earth System Model (CESM) of the National Center for Atmospheric Research (NCAR) on the basis of the Representative Concentration Pathway (RCP; [37]) 8.5 scenario for the period 2006–2100. Within an East Asian domain (104°E–144°E, 20°N–50°N), to gain the fine resolution grid dataset, boundary data from the RCP simulations were used to drive a regional climate model, the Weather Research and Forecasting (WRF) v3.5, which covers the domain with a 12.5 km×12.5 km resolution grid mesh. We performed the dynamical downscaling for two 20-year time slices, 2016–2035 and 2046–2065, in daily time-scale. In addition, the *Ts* data in a historical WRF run for the period 1979–2005 driven by the CESM simulation with historical greenhouse gas concentrations was used as the reference to project future changes in *Ts* compared to the present-day values (S1 Fig).

To identify the temporal and spatial varying trends in the LCD and *Ts* for 1989–2007, we calculated linear regression slopes of these two variables at each station and their distribution slope along latitude and elevation. Also, we calculated the correlation coefficient between the interannual variabilities of the mean LCD of the two tree species and the mean *Ts* averaged for a period from the mean LCD to some daily forward time step over the 54 stations to find the critical period when the variations of LCD and *Ts* are highly correlated. Dai et al. [33] suggested that the regression slopes between variations of autumn phenology and temperatures can be regarded as temperature sensitivity (days °C^-1^) for phenological events of tree species. We followed Dai et al. [33] to calculate the temperature sensitivities of the two tree species for each station. Finally, based on the observed relationship between the LCD and the *Ts*, we used future *Ts* dataset from a dynamic climate model, to predict future changes in LCD in response to *Ts* increases.

### Process-based model

Future changes in LCD in South Korea were projected using a process-based model, temperature-photoperiod model suggested by Jeong and Medvigy [30]. The temperature-photoperiod model partially reflects some physiological mechanism of leaf coloring; the initiation of leaf coloring may be affected by the accumulation of low temperature exposure and photoperiod triggering [28, 29]. Since the temperature-photoperiod model uses regional temperatures and photoperiods as inputs, it can be easily applied to various regions. The calculation procedure of the model is as follow:

$$\mathrm{CDD}(t)=\sum_{\mathrm{photoperiod}<P_{s}}^{t}\min(T_{i}\text{-}T_{b},0),$$ (1)

$$t=\text{leaf coloring date},\;\text{if CDD}(t)>{\text{CDD}}_{\text{threshold}}$$ (2)

where *P*_*s*_ and *T*_*b*_ refer to the standard photoperiod, the reference time point for accumulation and the base temperature of tree species related to the initiation of leaf coloring over the 54 weather stations, respectively, and *T*_*i*_ is the daily mean temperature. Cooling degree day, CDD(t), indicates the cooling degree accumulated from the time point when the photoperiod is below *P*_*s*_ to the time point of t. The time point when CDD(t) exceeds a certain threshold CDD_threshold_ is defined as the date of initiation of leaf coloring.

### Model fitting and validation for the two tree species

To fit the temperature-photoperiod model to the environmental conditions in South Korea, the data at the 54 stations are temporally divided into two groups. The data in the odd and even years are separately grouped for fitting and validation sets, respectively. The method using all station's dataset can be offset the site-specific biases such as the soil- and nutrient conditions [38]. Using the *Ts* and LCD data from the fitting set, we obtained 18361 parameter sets of the standard photoperiod (*P*_*s*_) and the base temperature (*T*_*b*_) by decreasing the standard photoperiod from 16 h to 10 h at 0.1 h intervals and increasing the base temperature from 5°C to 35°C at 0.1°C intervals. Corresponding threshold values (CDD_threshold_) were calculated for each standard photoperiod, base temperature, and the mean value of the LCD for 1989–2007 over the 54 stations. Finally, the parameter set yielding the smallest root mean square error (RMSE) with respect to the observed LCDs was selected as the best parameter set (Table 2).

**Table 2:** Best parameters of the temperature-photoperiod model for the two tree species in South Korea.

Standard photoperiod	Corresponding date	Base temperature	Threshold
(hours)	(day of year)	(°C)	(°C)
12.2	256	27.5	-446.4

To assess the model performance, we tested the results of each procedure through the general statistical method, modelling efficiency (ME).

$$\text{ME}=1\text{-}\frac{\sum_{\text{i}=1}^{\text{n}}{\left(O_{i}\;\text{-}\;P_{i}\right)}^{2}}{\sum_{\text{i}=1}^{\text{n}}{\left(O_{i}\;\text{-}\;{\bar{O}}_{i}\right)}^{2}},$$ (3)

where *O*_*i*_ and *P*_*i*_ represent observations and model predictions, respectively, and n is the number of component.

## Results

### Spatial-temporal characteristics of leaf coloring date and temperature

**Fig 1:**
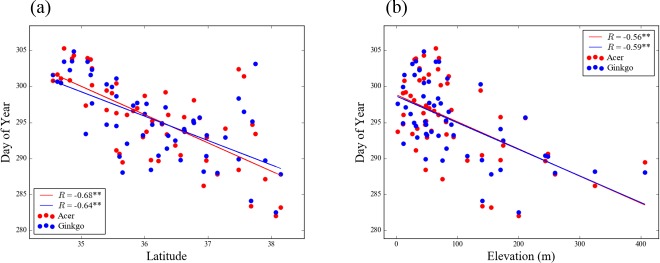
(a) Latitude and (b) elevation distributions of the mean leaf coloring date of the two tree species over the 54 stations. Red and blue solid lines depict the linear regression line of Acer and Ginkgo. R and ** symbol represent correlation coefficient and P < 0.01, respectively.

Fig 1 shows the latitude and elevation distributions of the mean LCD for the 54 stations. The mean LCD shows significantly negative correlations with both latitudes and elevations. Correlation coefficients (*R*) of -0.68 (-0.56) and -0.64 (-0.59) between LCD and latitude (elevation) are obtained at the 99% confidence level for *Acer* and *Ginkgo*, respectively. These latitudinal/elevational variations in LCD in South Korea are similar to those found in Japan [34]. Several outliers, which are the mean LCD exceeding 300 day of year (DOY) between 37.5°N and 38°N, were recorded at weather stations located in megacity, Seoul and Incheon, may suggest urbanization effects on LCD (Fig 1). Noodén and Schneider [39] suggested that streetlight of urban area can delay leaf coloring.

**Fig 2:**
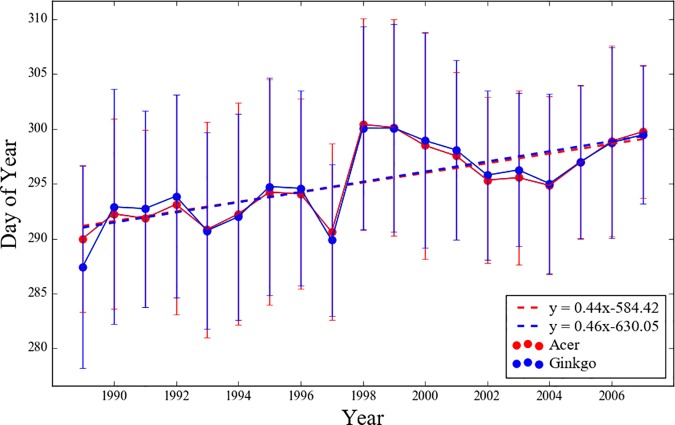
Interannual variations of the mean leaf coloring date of the two tree species at the 54 stations. Error bars indicate ±1 standard deviation. Dash line depict linear regression line of the mean leaf coloring date of the two tree species.

Fig 2 shows the interannual variations in the mean LCD and ±1 standard deviation over the 54 stations. It is noteworthy that the mean variations for the two tree species are very close (*R =* 0.91, *P* < 0.01) and have the same mean value of 295 DOY. This implies the presence of dominant common factors such as temperature, soil moisture, or precipitation, that affect the timing of LCD regardless of the connatural characteristics of each tree species. Among those, previous studies found that temperature is the major factor [12, 40]. The linear regression slope of the mean LCD for *Acer* and *Ginkgo* is also similar at 0.44 and 0.46 (i.e., about 0.45) days year^-1^, respectively, i.e., the dates have been delayed by about 8.4 and 8.7 days during the 19-year observation period. The similarity between these two tree species yields the same best parameter set in fitting the temperature-photoperiod model (Table 2). The significant shift point was detected between 1997 and 1998. The seasonal temperature was significantly higher in 1998 than in preceding years across East Asia [41]. Phenological events in other regions of East Asia are also affected by warm temperatures during this period. For example, an abrupt advancement of spring phenological events occurred in China between 1997 and 1998 [42].

**Fig 3:**
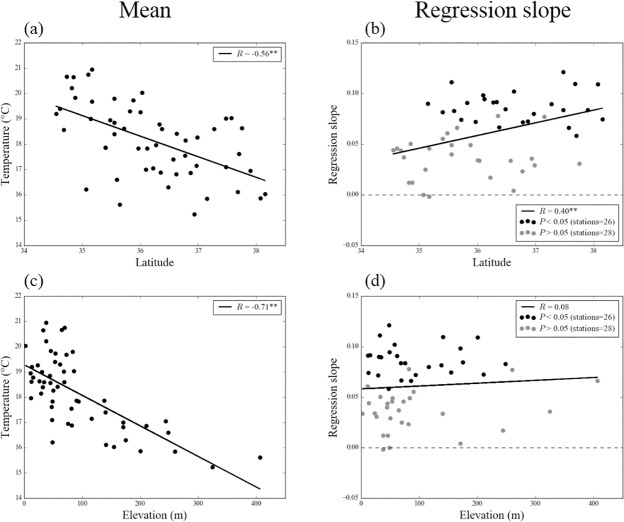
Latitude and elevation distributions for the mean (a) (c) and regression slope (b) (d) (°C per year) of the surface air temperature averaged from September 1st to the mean leaf coloring date in South Korea (295 DOY) during 1989 to 2007 in each stations. Black and grey color indicate significant and insignificant results at the 95% confidence level, respectively. *R* and ** symbol represent correlation coefficient and *P* < 0.01, respectively.

South Korea has been experiencing dramatic climate changes especially in temperatures [43]. Before analyzing the relationship between LCD and *Ts*, we examined the changing trend of the mean *Ts* averaged over September 1st to the mean LCD in South Korea (295 DOY) at the 54 stations (Fig 3). The results show that the positive *Ts* trends exist at most stations, and that there is a significant positive correlation between the changing trend of *Ts* and latitudes; however, its relationship with elevation is insignificant. Namely, the increase of *Ts* at colder stations are faster than those at warmer stations.

**Fig 4:**
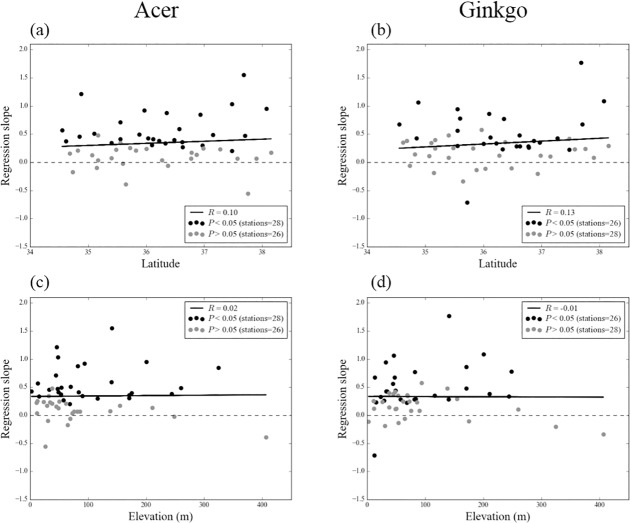
(a) (b) Latitude and (c) (d) elevation patterns of regression slope (days per year) of the LCD for two tree species during 1989–2007 in each stations. Black and grey color indicate significant and insignificant results at the 95% confidence level, respectively. Solid line depict the linear regression line using total 54 station's data. *R* represents correlation coefficient.

The positive regression slopes of *Ts* for latitude and elevation is seemed to be correlated with the changing trend and spatial patterns of the LCD. Linear regression slopes of the LCD (days per year) are positive for most of these stations (Fig 4), suggesting that the LCD in South Korea had been delayed during the period 1989–2007 at most stations. Although there is significant latitudinal pattern of the changing *Ts* trend at the 54 weather stations (Fig 3), no statistically significant latitudinal and elevational dependencies are found in the case of LCD, especially for elevations (Fig 4). Small positive *R* larger than 0.1 is the only indication that uncertain latitudinal variations in the LCD delaying at cold stations may be faster than those at warm stations (Fig 4).

### Relationship between leaf coloring dates and temperatures

**Fig 5:**
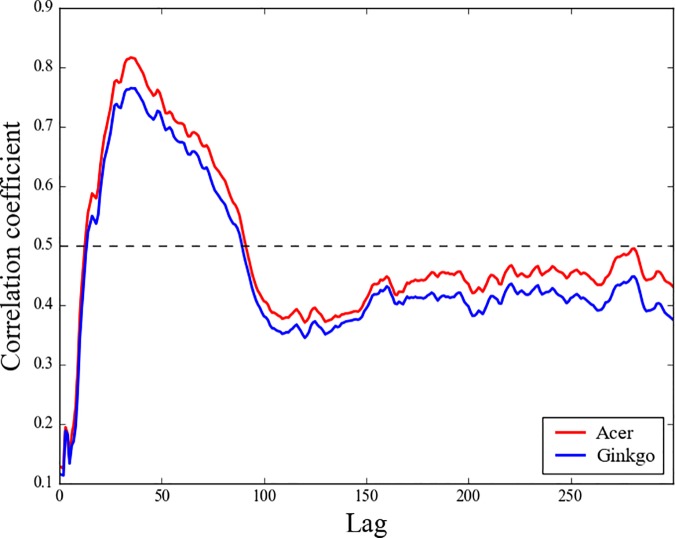
Correlations between interannual variations of the mean leaf coloring date of the two tree species and the mean surface air temperature averaged from 295 DOY to some forward lagged period.

The temperature variation averaged from the starting date of autumn phenological events to some prior date is closely correlated with the changes in those events [15, 16, 44]. Fig 5 shows the temperature-phenology relationship. Because the two tree species show very similar variability (Fig 2), their relationship with *Ts* is also similar. It shows close relationship with the variations of *Ts* in autumn and late summer (*R* > 0.7, *P* < 0.01). Extracting the point of the highest correlation, we found that the changes in the LCD of the two tree species in South Korea are closely correlated with the variation in *Ts* during 260–295 DOY (*R* = 0.82 in *Acer* and *R* = 0.77 in *Ginkgo*, *P* < 0.01). This period is also relatively well correlated with the LCD at individual stations (S2 Fig). It was suggested that the start of leaf coloring of *Acer* and *Ginkgo* in Japan is highly correlated with the change in the temperature averaged over the months of phenology to the preceding two months [34, 40]. We speculate that the difference between South Korea and Japan may be due to different local environmental conditions.

**Fig 6:**
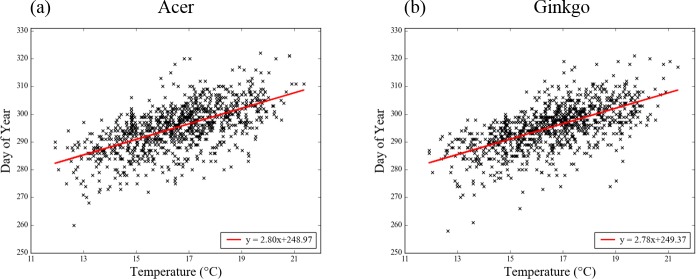
The scatter plot between the leaf coloring date of the two tree species and the mean surface air temperature averaged during 260–295 DOY for the total dataset. Red solid line depicts linear regression line.

The scatter plots of LCD and the mean *Ts* averaged over 260–295 DOY at individual stations and years (Fig 6) clearly indicate that the delaying of the LCD is related with *Ts* increases in mid-autumn (260–295 DOY). The linear regression slopes between LCD and the mean *Ts* for during 260–295 DOY (i.e., temperature sensitivity, days °C^-1^ [33]) are the same at about 2.8 days °C^-1^ at the 99% confidence level. Thus, the *Ts* variation for 260–295 DOY can play a decisive role in determining the LCD of the two tree species.

**Fig 7:**
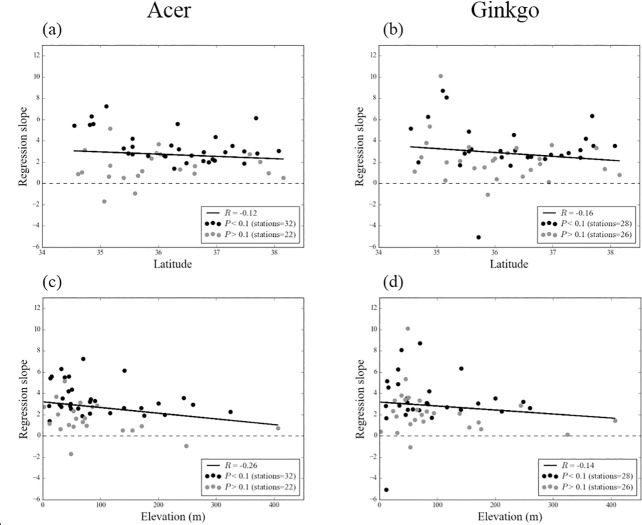
Same as Fig 4, but for regression slope (days per °C) between leaf coloring date of the two tree species and the mean surface air temperature averaged during 260–295 DOY in each stations. Black and grey color indicate significant and insignificant results at the 90% confidence level, respectively. *R* represents correlation coefficient.

To examine the effect of *Ts* on the latitude and elevation patterns of the LCD delaying, the temperature sensitivities at each stations are shown in Fig 7. The general latitude and elevation patterns of the temperature sensitivity are unclear; however, some negative patterns may exist. We suspect that it may slightly offset strong positive latitudinal patterns of *Ts* (Fig 3) to latitudinal patterns of the LCD delaying as seen in Fig 4. However, because analyzed latitudinal patterns of the temperature sensitivity and the LCD delaying are below the statistical significant level of 90%, it may come from some random effects caused by manually observed dataset. The effects of other factors at individual stations may need to be considered in determining the LCD.

### Assessment of simulating performances of the temperature-photoperiod model

**Fig 8:**
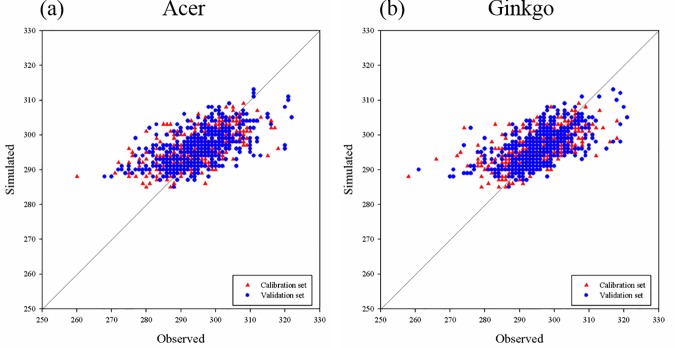
The scatter plot for leaf coloring date of the two tree species between observations and model simulations for the calibration set (red triangle) and validation set (blue circle).

The performance of the temperature-photoperiod model must be evaluated before future projections for reliable projections. We simulated the LCD from 1989 to 2007 using the temperature-photoperiod model with the best parameter set obtained from the fitting procedure using the *Ts* and photoperiod datasets at the 54 stations. We assessed the performance of the model for each procedure by comparing model results against the observed LCD (Table 3). Relatively small RMSE and biases in conjunction with high ME compared to observations imply that the temperature-photoperiod model, in conjunction with the best parameter set, performs well in simulating the LCD of *Acer* and *Ginkgo* in South Korea. Comparison of the simulated and observed LCD at the 54 stations (Fig 8) shows that the majority of data points are concentrated along a diagonal line, indicating close agreements between the modelled and observed values. This evaluation supports the capability of the temperature-photoperiod model, which can well simulate the climatology of the leaf coloring date close to observations even if its performance of intra-decadal simulation is relatively low. In addition, the simulated mean LCD over the 54 stations agrees well with observations (*R* > 0.75; *P* < 0.01) and the differences between the simulated and observed dates are less than 5 days (S3 Fig). In particular, the large fluctuations of the mean LCD for the two years, 1997 and 1998, are well replicated in the simulation. Consequently, we expect that the LCD predictions from the temperature-photoperiod model can provide useful insights into future changes. In addition, the close relationship between the variations of LCD and *Ts* is confirmed using the temperature-photoperiod model which assumes that the process of photoperiod and temperature accumulation triggers leaf coloring.

**Table 3:** The results of the temperature-photoperiod model assessment. n is the number of dataset.

Acer				Ginkgo			
	ME	RMSE	Bias		ME	RMSE	Bias
Fitting	0.36	6.56	-1.01	Fitting	0.37	6.41	-1.05
(n = 521)				(n = 525)			
Validation	0.32	7.09	-0.96	Validation	0.34	6.73	-0.77
(n = 478)				(n = 480)			
Total	0.34	6.82	-0.99	Total	0.36	6.57	-0.92
(n = 999)				(n = 1005)			

To further investigate the model performance and its relationship with the effects of photoperiod and *Ts*, we applied the process-based model of Delpierre et al. [28], which can be able to disentangle the relative effects of the *Ts* and photoperiod on process of leaf coloring separately. The results showed that the effects of *Ts* and photoperiod are represented in the same way both in the temperature-photoperiod model and the model of Delpierre et al. [28]. We also tested the temperature-photoperiod model by considering a fixed starting date for the initiation of the temperature accumulation, which has no influence of the photoperiod. The results indicated that using both factors are better than using only temperatures; biases and RMSE are higher and ME is lower at considering the temperature only. These imply that the effects of photoperiod may be deeply involved in the process of leaf coloring in South Korea as much as the *Ts* and temperature-photoperiod model can replicate and simulate their close relations.

### Projection of future changes in leaf coloring date

**Fig 9:**
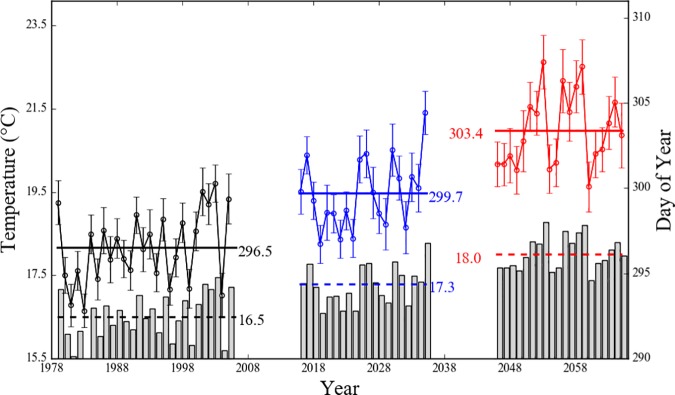
Interannual variations of the mean leaf coloring date (symbols with solid line) and the mean surface air temperature (gray bar) averaged from the date at photoperiod less than best parameter (i.e., 12.2 h) to leaf coloring date predicted by a temperature-photoperiod model with WRF dataset. Horizontal solid and dashed lines represent the mean value of the leaf coloring date and surface air temperature for each periods, respectively. Error bars indicate confidence interval at the 95% confidence level.

Future LCDs were projected for the RCP 8.5 WRF *Ts* data for 2016–2035 and 2046–2065 using the temperature-photoperiod model in conjunction with the best parameter set. We also simulated past LCDs using historical WRF *Ts* data for 1979–2005 as the reference for the projection. These calculations were performed at the 54 points nearest to each weather stations using the forcing values averaged over the nearest nine grids points. Resulting interannual variations in the mean LCD and the mean *Ts* averaged over the period from the photoperiod is less than 12.2 h to the day the accumulated temperature exceeds the threshold value (i.e., simulated LCD) as indicated in the best parameter set (Table 2) are shown in Fig 9. For the past period from 1979 to 2005, although the intra-decadal LCD variability is somewhat different from the observations as seen in Fig 2, the mean value is almost the same as for 296.5 DOY. Considering it is impossible to perfectly replicate the real environment in climate models, researchers commonly focused on climatological agreement between observations and model simulations to ensure the reliability of future predictions [45, 46, 47]. In this respect, the about one-day difference in the LCD between observations and model predictions suggest that the projections for future LCD are reliable.

As *Ts* gradually increases, future LCDs are delayed. It is noteworthy that although their intra-decadal variability is large, the mean LCD in each future period show intensification of differences between periods; from 1979–2005 (2016–2035) to 2016–2035 (2046–2065) the mean LCD would be delayed for 3.2 (3.7) days. However, because the projections in this study are only based on the temperature-photoperiod model and the *Ts* predictions was simulated by an one climate model, it should be considered as a possibility of future changes in LCD in response to *Ts* changes.

## Discussion

Using long-term observations over a large area in South Korea, this study has found that the LCD of *Acer palmatum* and *Ginkgo biloba* in South Korea has been delayed by increases in *Ts* in mid-autumn similarly as other deciduous tree species in Europe and North America, for example, *Acer rubrum* and *Quercus velutina* in North America [15, 30], and *Fagus sylvatica* and *Quercus rubra* in France and Germany [16, 28, 29]. These close relationships between temperatures and leaf coloring in South Korea suggest that the effect of the changes in temperature is the primary factor to the recent autumn phenological changes in East Asia. Of course, effects of other factors also should be considered [48].

Although the effects of photoperiod to autumn phenology was importantly dealt with in previous literatures, a recent study suggested that its role in the determining the timing of autumn phenology and in the phenological model seems to be not decisive [49]. However, we found that photoperiod is involved the leaf coloring process of tree species in South Korea, and we can disentangle its effect from the effect of temperature through using process-based model. This may be because the photoperiod is powerful regulator to autumn phenology in the region with severe winter or high latitude [24]. South Korea has relatively severe winter compared to other regions in similar latitudes due to generally affected by northern cold air from Siberia in winter season [50].

In addition to temperatures and photoperiods, precipitation also plays a critical role in shifting vegetation phenology in dry regions where vegetation growth is limited by water availability [51, 52, 53]. South Korea generally experiences wet conditions lasting for over four months from the first and second rainy season before leaf coloring starts [54, 55]. It is possible that droughts will occur, hence, future studies are needed to examine the effect of precipitation (and soil moisture) using accurate drought assessment tools or methods. In addition, recent studies suggested that the spring phenology may affect the timing of autumn phenological events [56, 57]. Since KMA does not observe the spring phenological events of *Acer* and *Ginkgo*, the relationship between the spring and autumn phenological events is not explored in this study.

Spatial variations in the mean LCD for *Acer* and *Ginkgo* in South Korea for the period 1989–2007 show significantly negative correlations with latitudes and elevations like in Japan [34]. However, resulting temperature sensitivity is somewhat ambiguous compared to the study of Doi and Takahashi [34]. These two tree species in Japan show significant negative relationships between the temperature sensitivity and latitudes, while those in South Korea show a weak negative, but insignificant, relationship. These suggest that tree species may respond differently to temperatures according to the environmental conditions of their habitat due to local adaptation. Thus, different responses can be detected for the same species living in different regions. This presents the importance of analyzing species-specific phenological responses within the framework of local conditions such as climate and soil characteristics. Further, it implies that the parameterized autumn phenology in general circulation models must consider local phenological responses to each environmental factor even though they deal with the same species. In this respect, this study will contribute to enhancement of modelling skill to predict autumn phenology in East Asia.

To predict future changes in LCD in response to *Ts* increases, we used a temperature-photoperiod model, a process-based model which embodies the effect of temperature and photoperiod to leaf development processes. Although the process-based model can simulate the observed LCD variations reasonably, obvious limitations must be discussed as well. Except the effects of photoperiod and temperature, other environmental factors may need to be included in simulating process of model. For example, the large vapor pressure deficit during a growing season can slowdown photosynthetic activities that, in turn, can affect the timing of phenological events in the following autumn [58]. Also, the potential effects of high temperatures exceeding 35°C may damage the photosynthetic apparatus [59]. Fundamentally, in the some observations, the rate of the change in phenological events is getting slower, perhaps because it has reached certain phenological threshold [60]. However, present process-based models cannot include such recent changing trends. Nevertheless, it is encouraging that the process-based model driven by only photoperiod and temperature has performed well in simulating autumn phenology with small uncertainties in key aspects such as the differences in the inherited traits between species, inhomogeneous local environmental conditions, and genetic acclimation [28, 29, 30]. Thus, we expect that the LCD predictions for South Korea from the temperature-photoperiod model can reliably project future phenology.

A hybrid approach in which a temperature-photoperiod model is used in conjunction with a regional climate model, shows that continued delaying of LCD is expected in the future in response to gradual *Ts* increases. We expect that the delaying will proceed together with increases in temperature sensitivities of LCD in the future period. In a warmer climate, the cold-degree requirement for initiating leaf coloring would need longer duration of heat exposure than in colder climate. This in turn implies that tree species in future periods will be exposed to relatively warm temperatures for longer periods than in the present climate [33]. Accordingly, the temperature sensitivity may increase in warmer conditions.

Results presented herein are noteworthy, as they represent the first attempt to investigate both past and future changes in leaf coloring in South Korea using long-term observations covering a wide area and fine-resolution climate model data. Although there are limitations in future projections, perhaps due to the uncertainties of using single regional climate model, climate change scenario and process-based model, their reliability can be supported by the general agreement with previous studies for other regions. In conclusion, the quantitative projections presented in this study show the approaching changes in leaf coloring that cannot be neglected, which may put pressure on forestry policy makers to prepare for climate changes in South Korea.

## Supporting information

S1 Fig Scatter plot of the daily temperature of the 54 station values versus WRF grid values which are closest to the 54 stations for 1989–2005. (TIF)

S2 Fig The highest correlation period for the leaf coloring date of (a) Acer and (b) Ginkgo to the surface air temperature in each stations. Red, gray, and blue indicate correlation coefficient greater than 0.5, between 0.5 and 0.0, and lower than 0.0, respectively. Dashed lines represent the period from 260 to 295 DOY.
(TIF)

S3 Fig Interannual variations of observed (black) and model simulated (red) mean leaf coloring date of the two tree species. Gray shaded area and error bars indicate ±1 standard deviation of observations and model simulations, respectively. *R* and ** symbol represent correlation coefficient and *P* < 0.01, respectively.
(TIF)

S1 File The raw phenology dataset recorded by KMA (in Korean). (ZIP)

S2 File The WRF surface air temperature dataset made by Climate Physics Laboratory in Seoul National University. (ZIP)

## References

[1] Walther GR, Post E, Convey P, Menzel A, Parmesan C, Beebee TJ, et al (2002) Ecological responses to recent climate change. Nature 416: 389–395. 10.1038/416389a 11919621

[2] Zhang N, Shugart H, Yan X (2009) Simulating the effects of climate changes on eastern Eurasia forests. Climatic Change 95: 341–361.

[3] Jeong SJ, Ho CH, Gim HJ, Brown ME (2011) Phenology shifts at start vs. end of growing season in temperate vegetation over the Northern Hemisphere for the period 1982–2008. Global Change Biology 17: 2385–2399.

[4] Piao S, Friedlingstein P, Ciais P, Viovy N, Demarty J (2007) Growing season extension and its impact on terrestrial carbon cycle in the Northern Hemisphere over the past 2 decades. Global Biogeochemical Cycles 21: GB3018,

[5] Bonan GB (2008) Forests and climate change: Forcings, feedbacks, and the climate benefits of forests. Science 320: 1444–1449. 10.1126/science.1155121 18556546

[6] Richardson AD, Keenan TF, Migliavacca M, Ryu Y, Sonnentag O, Toomey M (2013) Climate change, phenology, and phenological control of vegetation feedbacks to the climate system. Agricultural and Forest Meteorology 169: 156–173.

[7] Chuine I, Beaubien EG (2001) Phenology is a major determinant of tree species range. Ecology Letters 4: 500–510.

[8] Klanderud K (2005) Climate change effects on species interactions in an alpine plant community. Journal of Ecology 93: 127–137.

[9] Chmura DJ, Anderson PD, Howe GT, Harrington CA, Halofsky JE, Peterson DL, et al (2011) Forest responses to climate change in the northwestern United States: Ecophysiological foundations for adaptive management. Forest Ecology and Management 261: 1121–1142.

[10] Menzel A, Fabian P (1999) Growing season extended in Europe. Nature 397: 659–659.

[11] Chen XQ, Hu B, Yu R (2005) Spatial and temporal variation of phenological growing season and climate change impacts in temperate eastern China. Global Change Biology 11: 1118–1130.

[12] Ho CH, Lee EJ, Lee I, Jeong SJ (2006) Earlier spring in Seoul, Korea. International Journal of Climatology 26: 2117–2127.

[13] Jeong JH, Ho CH, Linderholm HW, Jeong SJ, Chen D, Choi YS (2011) Impact of urban warming on earlier spring flowering in Korea. International Journal of Climatology 31: 1488–1497.

[14] Shen M, Sun Z, Wang S, Zhang G, Kong W, Chen A, Piao S (2013) No evidence of continuously advanced green-up dates in the Tibetan Plateau over the last decade. Proceedings of the National Academy of Science of the United States of America, 110, E2329. 10.1073/pnas.1304625110 PMC3696789 23661054

[15] Archetti M, Richardson AD, O'Keefe J, Delpierre N (2013) Predicting climate change impacts on the amount and duration of autumn colors in a New England forest. PLOS ONE 8: e57373, 10.1371/journal.pone.0057373 23520468 PMC3592872

[16] Estrella N, Menzel A (2006) Responses of leaf colouring in four deciduous tree species to climate and weather in Germany. Climate Research 32: 253.

[17] Zhu W, Tian H, Xu X, Pan Y, Chen G, Lin W (2012) Extension of the growing season due to delayed autumn over mid and high latitudes in North America during 1982–2006. Global Ecology and Biogeography 21: 260–271.

[18] Garonna I, Jong R, Wit AJ, Mücher CA, Schmid B, Schaepman ME (2014) Strong contribution of autumn phenology to changes in satellite-derived growing season length estimates across Europe (1982–2011). Global Change Biology 20: 3457–3470. 10.1111/gcb.12625 24797086

[19] Piao S, Ciais P, Friedlingstein P, Peylin P, Reichstein M, Luyssaert S, et al (2008) Net carbon dioxide losses of northern ecosystems in response to autumn warming. Nature 451: 49–52. 10.1038/nature06444 18172494

[20] Wu CY, Gonsamo A, Chen JM, Kurz WA, Price DT, Lafleur PM, et al (2012) Interannual and spatial impacts of phenological transitions, growing season length, and spring and autumn temperatures on carbon sequestration: A North America flux data synthesis. Global and Planetary Change 92–93: 179–190.

[21] Schaber J, Badeck FW (2003) Physiology-based phenology models for forest tree species in Germany. International Journal of Biometeorology 47: 193–201. 10.1007/s00484-003-0171-5 12698325

[22] Koike T (1990) Autumn coloring, photosynthetic performance and leaf development of deciduous broad-leaved trees in relation to forest succession. Tree Physiology 7: 21–32. 14972903 10.1093/treephys/7.1-2-3-4.21

[23] Lee DW, O'Keefe J, Holbrook NM, Feild TS (2003) Pigment dynamics and autumn leaf senescence in a New England deciduous forest, eastern USA. Ecological Research 18: 677–694.

[24] Estiarte M, Peñuelas J (2015) Alteration of the phenology of leaf senescence and fall in winter deciduous species by climate change: Effects on nutrient proficiency. Global Change Biology 21: 1005–1017. 10.1111/gcb.12804 25384459

[25] Yu R, Schwartz MD, Donnelly A, Liang L (2016) An observation-based progression modeling approach to spring and autumn deciduous tree phenology. International Journal of Biometeorology 60: 335–349. 10.1007/s00484-015-1031-9 26219605

[26] Lebourgeois F, Pierrat JC, Perez V, Piedallu C, Cecchini S, Ulrich E (2010) Simulating phenological shifts in French temperate forests under two climatic change scenarios and four driving global circulation models. International Journal of Biometeorology 54: 563–581. 10.1007/s00484-010-0305-5 20300777

[27] Vitasse Y, Portê AJ, Kremer A, Michalet R, Delzon S (2009) Responses of canopy duration to temperature changes in four temperate tree species: Relative contributions of spring and autumn leaf phenology. Oecologia 161: 187–198. 10.1007/s00442-009-1363-4 19449036

[28] Delpierre N, Dufrêne E, Soudani K, Ulrich E, Cecchini S, Boê J, et al (2009) Modelling interannual and spatial variability of leaf senescence for three deciduous tree species in France. Agricultural and Forest Meteorology 149: 938–948.

[29] Vitasse Y, François C, Delpierre N, Dufrêne E, Kremer A, Chuine I, et al (2011) Assessing the effects of climate change on the phenology of European temperate trees. Agricultural and Forest Meteorology 151: 969–980.

[30] Jeong SJ, Medvigy D (2014) Macroscale prediction of autumn leaf coloration throughout the continental United States. Global Ecology and Biogeography 23: 1245–1254.

[31] IPCC (2014) Fifth Assessment Report. Cambridge: Cambridge University Press.

[32] Ibáñez I, Primack RB, Miller-Rushing AJ, Ellwood E, Higuchi H, Lee SD, Kobori H, Silander JA (2010) Forecasting phenology under global warming. Philosophical Transactions of the Royal Society of London B: Biological Sciences 365: 3247–3260. 10.1098/rstb.2010.0120 20819816 PMC2981942

[33] Dai J, Wang H, Ge Q (2014) The spatial pattern of leaf phenology and its response to climate change in China. International Journal of Biometeorology 58: 521–528. 10.1007/s00484-013-0679-2 23732443

[34] Doi H, Takahashi M (2008) Latitudinal patterns in the phenological responses of leaf colouring and leaf fall to climate change in Japan. Global Ecology and Biogeography 17: 556–561.

[35] Ge Q, Dai J, Zheng J, Bai J, Zhong S, Wang H, et al (2011) Advances in first bloom dates and increased occurrences of yearly second blooms in eastern China since the 1960s: Further phenological evidence of climate warming. Ecological Research 26: 713–723.

[36] Xu L, Chen X (2013) Regional unified model‐based leaf unfolding prediction from 1960 to 2009 across northern China. Global Change Biology 19: 1275–1284. 10.1111/gcb.12095 23504902

[37] Moss RH, Edmonds JA, Hibbard KA, Manning MR, Rose SK, van Vuuren DP, et al (2010) The next generation of scenarios for climate change research and assessment. Nature 463: 747–756. 10.1038/nature08823 20148028

[38] Winder M, Cloern JE (2010) The annual cycles of phytoplankton biomass. Philosophical Transactions of the Royal Society of London B: Biological Sciences 365: 3215–3226. 10.1098/rstb.2010.0125 20819814 PMC2981943

[39] Noodén LD, Schneider MJ (2004) Light control of senescence In: Noodén LD (Ed.), Plant Cell Death Processes. Academic Press.

[40] Matsumoto K, Ohta T, Irasawa M, Nakamura T (2003) Climate change and extension of the Ginkgo biloba L. growing season in Japan. Global Change Biology 9: 1634–1642.

[41] Bell GD, Halpert MS, Ropelewski CF, Kousky VE, Douglas AV, Schnell RC, Gelman ME (1999) Climate assessment for 1998. Bulletin of the American Meteorological Society 80: S1–S48.

[42] Piao S, Fang J, Zhou L, Ciais P, Zhu B (2006) Variations in satellite‐derived phenology in China's temperate vegetation. Global Change Biology 12: 672–685.

[43] Jung HS, Choi Y, Oh JH, Lim GH (2002) Recent trends in temperature and precipitation over South Korea. International Journal of Climatology 22: 1327–1337.

[44] Chen X, Xu L (2012) Phenological responses of Ulmus pumila (Siberian Elm) to climate change in the temperate zone of China. International Journal of Biometeorology 56: 695–706. 10.1007/s00484-011-0471-0 21805230

[45] Hong SY, Moon NK, Lim KSS, Kim JW (2010) Future climate change scenarios over Korea using a multi-nested downscaling system: a pilot study. Asia-Pacific Journal of Atmospheric Science 46: 425–435.

[46] Park CK, Byun HR, Deo R, Lee BR (2015) Drought prediction till 2100 under RCP 8.5 climate change scenarios for Korea. Journal of Hydrology 526: 221–230.

[47] Yu E, Sun J, Chen H, Xiang W (2015) Evaluation of a high-resolution historical simulation over China: Climatology and extremes. Climate Dynamics 45: 2013–2031.

[48] Schwartz MD (2003) Phenology: An integrative environmental science. Springer: 564pp.

[49] Olsson C, Jönsson AM (2015) A model framework for tree leaf colouring in Europe. Ecological Modelling 316: 41–51.

[50] Min SK, Son SW, Seo KH, Kug JS, An SI, Choi YS, Jeong JH, Kim BM, Kim JW, Kim YH, Lee JY, Lee MI (2015) Changes in weather and climate extremes over Korea and possible causes: A review. Asia-Pacific Journal of Atmospheric Sciences 51: 103–121.

[51] Yang Y, Guan H, Shen M, Liang W, Jiang L (2015) Changes in autumn vegetation dormancy onset date and the climate controls across temperate ecosystems in China from 1982 to 2010. Global Change Biology 21: 652–665. 10.1111/gcb.12778 25430658

[52] Bowers JE (2007) Has climatic warming altered spring flowering date of Sonoran desert shrubs? The Southwestern Naturalist 52: 347–355.

[53] Jenerette GD, Scott RL, Huete AR (2010) Functional differences between summer and winter season rain assessed with MODIS‐derived phenology in a semi‐arid region. Journal of Vegetation Science 21: 16–30.

[54] Ho CH, Kang IS (1988) The variability of precipitation in Korea. Journal of Korean Meteorology Society 24: 38–48.

[55] Ho CH, Kim JH, Lau KM, Kim KM, Gong D, Lee YB (2005) Interdecadal changes in heavy rainfall in China during the northern summer. Terrestrial Atmospheric and Oceanic Sciences 16: 1163–1176.

[56] Fu YSH, Campioli M, Vitasse Y, De Boeck HJ, den Berge JV, Elgawad HA, et al (2014) Variation in leaf flushing date influences autumnal senescence and next year’s flushing date in two temperate tree species. Proceedings of the National Academy of Sciences of the United States of America 111: 7355–7360. 10.1073/pnas.1321727111 24799708 PMC4034254

[57] Keenan TF, Richardson A (2015) The timing of autumn senescence is affected by the timing of spring phenology: Implications for predictive models. Global Change Biology 21: 2634–2641. 25662890 10.1111/gcb.12890

[58] Bassow SL, Bazzaz FA (1998) How environmental conditions affect canopy leaf-level photosynthesis in four deciduous tree species. Ecology 79: 2660–2675.

[59] Berry J, Björkman O (1980) Photosynthetic response and adaptation to temperature in higher plants. Annual Review of Plant Physiology 31: 491–543.

[60] Iler AM, Hoye TT, Inouye DW, Schmidt NM (2013) Nonlinear flowering responses to climate: Are species approaching their limits of phenological change? Philosophical Transactions of the Royal Society B: Biological Sciences 368: 20120489. 10.1098/rstb.2012.0489 PMC3720060 23836793

