# Supplementary figures and images for: Spatial and temporal changes in leaf coloring date of Acer palmatum and Ginkgo biloba in response to temperature increases in South Korea
Source: PLoS One. 2017 Mar 27;12(3):e0174390. doi: 10.1371/journal.pone.0174390 (PMC5367789; doi:10.1371/journal.pone.0174390)

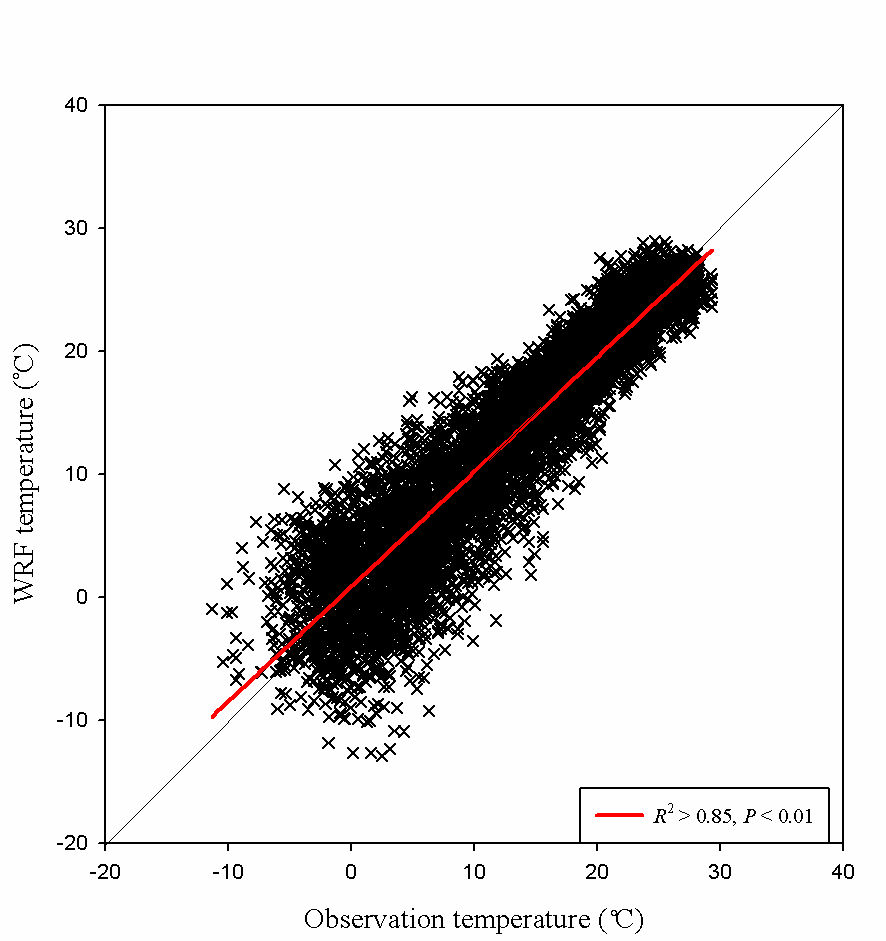

Supplement: S1 Fig — (TIF) [file pone.0174390.s001.tif]

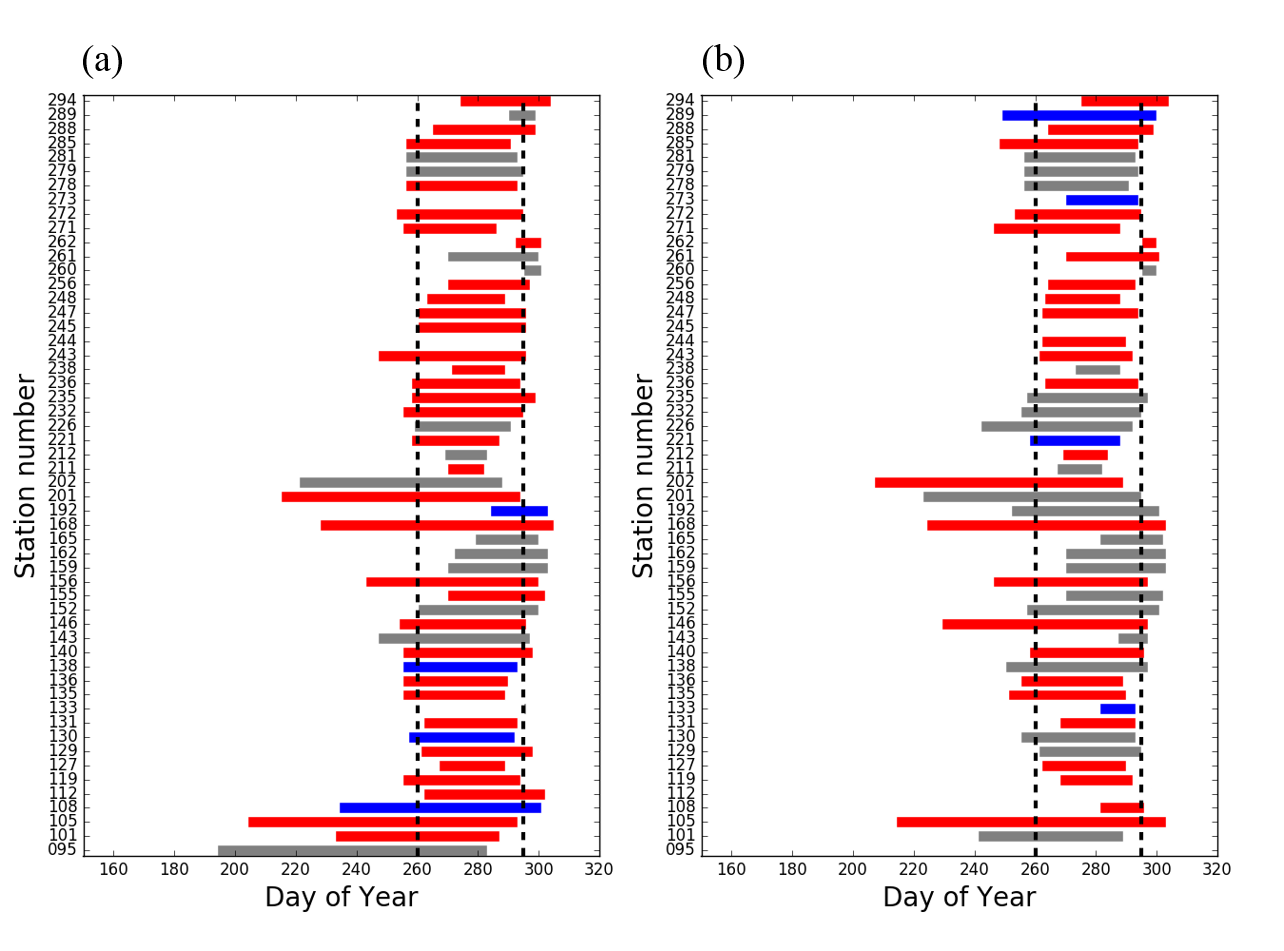

Supplement: S2 Fig — The highest correlation period for the leaf coloring date of (a) Acer and (b) Ginkgo to the surface air temperature in each stations. Red, gray, and blue indicate correlation coefficient greater than 0.5, between 0.5 and 0.0, and lower than 0.0, respectively. Dashed lines represent the period from 260 to 295 DOY. (TIF) [file pone.0174390.s002.tif]

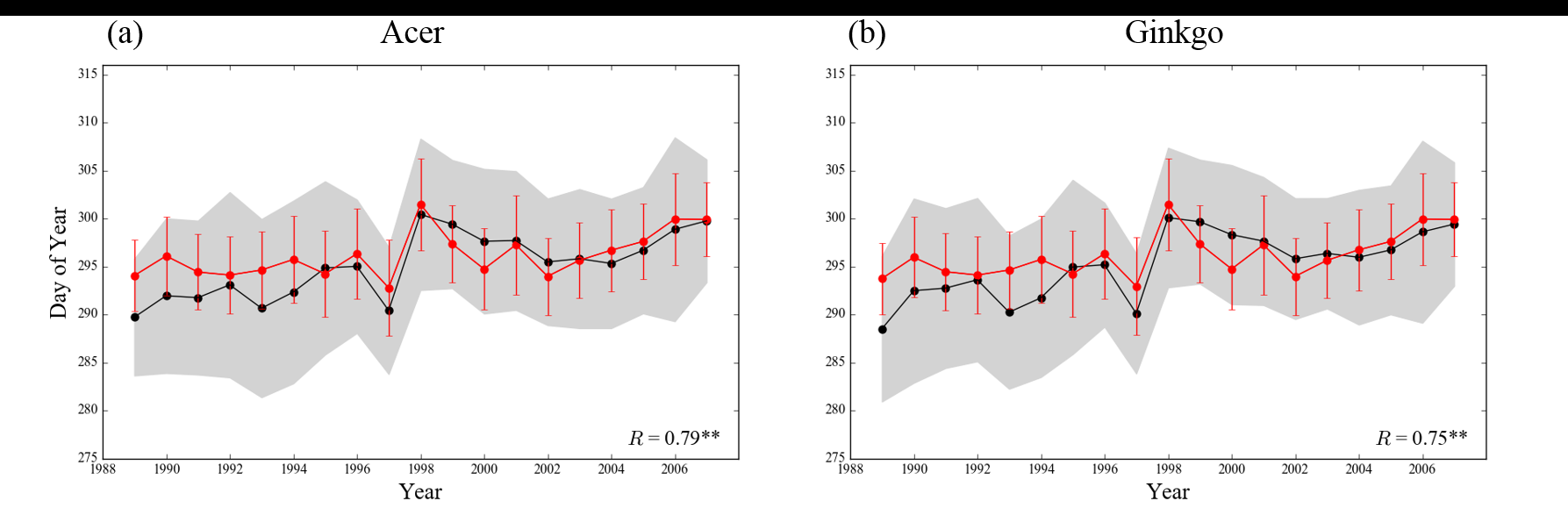

Supplement: S3 Fig — Gray shaded area and error bars indicate ±1 standard deviation of observations and model simulations, respectively. R and ** symbol represent correlation coefficient and P < 0.01, respectively. (TIF) [file pone.0174390.s003.tif]

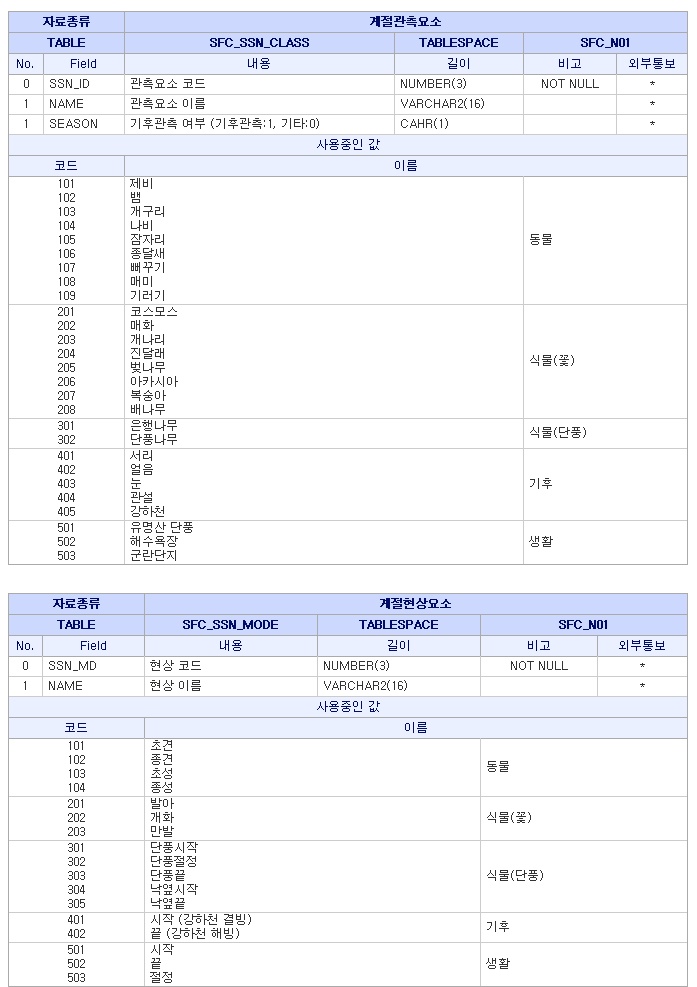

Supplement: S1 File — (ZIP) [file pone.0174390.s004.zip › Data_info.jpg]
